# Cytoskeletal architecture and its evolutionary significance in amoeboid eukaryotes and their mode of locomotion

**DOI:** 10.1098/rsos.160283

**Published:** 2016-09-28

**Authors:** Yonas I. Tekle, Jessica R. Williams

**Affiliations:** Spelman College, 350 Spelman Lane Southwest, Atlanta, GA 30314, USA

**Keywords:** cytoskeleton, amoeboid movement, microtubules, actin, immunocytochemistry, Amoebozoa

## Abstract

The cytoskeleton is the hallmark of eukaryotic evolution. The molecular and architectural aspects of the cytoskeleton have been playing a prominent role in our understanding of the origin and evolution of eukaryotes. In this study, we seek to investigate the cytoskeleton architecture and its evolutionary significance in understudied amoeboid lineages belonging to Amoebozoa. These amoebae primarily use cytoplasmic extensions supported by the cytoskeleton to perform important cellular processes such as movement and feeding. Amoeboid structure has important taxonomic significance, but, owing to techniques used, its potential significance in understanding diversity of the group has been seriously compromised, leading to an under-appreciation of its value. Here, we used immunocytochemistry and confocal microscopy to study the architecture of microtubules (MTs) and F-actin in diverse groups of amoebae. Our results demonstrate that all Amoebozoa examined are characterized by a complex cytoskeletal array, unlike what has been previously thought to exist. Our results not only conclusively demonstrate that all amoebozoans possess complex cytoplasmic MTs, but also provide, for the first time, a potential synapomorphy for the molecularly defined Amoebozoa clade. Based on this evidence, the last common ancestor of amoebozoans is hypothesized to have had a complex interwoven MT architecture limited within the granular cell body. We also generate several cytoskeleton characters related to MT and F-actin, which are found to be robust for defining groups in deep and shallow nodes of Amoebozoa.

## 1. Introduction

Unicellular eukaryotes that lack definitive shape and move by extension of cytoplasmic projections (pseudopodia—false feet) are commonly known as amoebae. While the amoeboid forms, shapes and movement types are widespread in the eukaryotic tree of life with the exception of plants, its origin and evolution is poorly understood [1,2]. There are generally four types of pseudopodia recognized in eukaryotes, which include axopodia (stiff microtubule (MT)-supported structure), filopodia (slender pointed ends), lobopodia (short and blunt finger-like structure) and reticulopodia (net-like structure). The majority of amoeboids with lobose type of pseudopodia (flat or cylindrical) fall under the supergroup Amoebozoa [3], the focus of this study. Interestingly, the Amoebozoa assemblage is primarily recovered from molecular genetic analysis without any known defining morphological or molecular synapomorphy [3–5]. Finding a unifying synapomorphy, using advanced techniques would aid in our understating of this group's evolution and biodiversity.

The taxonomy of Amoebozoa is one of the least understood among Protists. For over 200 years, morphology-based classification has failed in capturing the full diversity and phylogenetic relationships of amoeboid lineages [4,6–9]. Morphological characters that have been studied include general cell structure (shape, size, coat/test), mechanisms of locomotion and ultrastructural characters related to nucleus, mitochondrion and other subcellular structures (reviewed in [10]). Based on the technique used (light, electron microscope or molecular), a series of taxonomic systems have been established [4,8,11]. The usage of pseudopodial characters, obtained using simple light microscopy methods, has been challenged and remains controversial [12], whereas the utility of ultrastructural characters, especially those related to the fine structure of the nucleus, is mainly limited to lower (genus)-level classifications [4]. Early molecular phylogeny of amoebozoans revealed major discordance to the morphology-based classification schemes [13–15]. Subsequent molecular studies necessitated dramatic revisions followed by refinements and redefinition of some morphological features [10,16,17]. Interestingly, revised morphological characters related to pseudopodial structure and amoeboid movements have been shown to be more successful, and corroborated molecular phylogenies both at shallow and more inclusive nodes [4,5]. Based on molecular and morphological data, several clades, including subclades of diverse groups with morphological synapomorphy, have been recognized, though many higher-level groups remain unresolved [12]. Even though additional sampling of genes [12,18] and taxa [5,19] might help resolve relationships within Amoebozoa, defining anomalous relationships that drastically differ in morphology will require finding fine structural similarities to corroborate inferred molecular phylogenies [12]. So far, pseudopodial features hold the most promising morphological characters both at shallow and deeper nodes [10,12].

The phylogenetic utility of cytoskeletal features, particularly MTs, in eukaryotes with a locomotive organelle (flagellum or cilium) is well demonstrated [17,20–25]. Most amoebae have no specialized locomotive structures such as a cilium or flagellum (with the exception of the few amoeboflagellate taxa); their movement involves largely the entire cell. When in a stationary state, an amoeba can assume several shapes, often regarded as a formless blob. However, once in motion, distinct shapes (morphotypes) can be observed. Smirnov [26] noted 15 morphotypes based on the locomotive forms. Locomotive morphotypes in amoebae are identified based on those that are capable of forming pseudopodia, subpseudopodia and those that move as a whole cell. The outlines of morphotypes, individually or in combination, are useful in identifying amoebae, some of which are also found to be congruent with the molecular-based classification [4,12]. This clearly demonstrates that the formation of pseudopodia, cell shapes and movements in amoebae, are not entirely a random feature, but rather follow a pattern involving a complex subcellular process of cytoskeletal proteins and their interactions.

In *Amoeba proteus,* with a lobopodium type of pseudopodia, the microfilament system (actin and myosin) is the main driving force of amoeboid movement [26–29]. The system of MTs in *Amoeba proteus* is poorly developed and its role is not well understood [30,31]. In some members of the Amoebozoa, including *Acanthamoeba* [32], *Stygamoeba* [33] and *Stereomyxa* [34], the MT system appears more prominent in the form of a network and cytoplasmic microtubule-organizing centre (MTOC). These reports indicate that mechanisms of amoeboid locomotion differ among amoebozoans at the proteome-level.

Given the paucity and homoplasious nature of the current morphological data and lack of refined analysis of the most promising feature, i.e. pseudopodia, in amoebozoan taxonomy, it is critical to carefully examine attributes of amoeboid movement at the proteome level. Use of advanced techniques to study amoeboid structure is expected to yield finer details unrecoverable by simple light microscopy, thereby enabling a better homology assessment in the group. In this study, we used immunocytochemistry (ICC) and confocal fluorescence microscopy to study amoeboid shape and structure in diverse distantly related amoebae representing all known major subclades of Amoebozoa [10,12,18] and in closely related species belonging to the genus *Cochliopodium*. Our findings demonstrate that cytoskeletal characters obtained using ICC not only revealed fine structural differences useful for resolving relationships at shallow and deeper nodes, but also gave insights on the diversity and mechanisms of amoeboid movement employed in the Amoebozoa.

## 2. Results

### 2.1. Immunocytochemistry staining of microtubules in Amoebozoa

**Figure 1.**
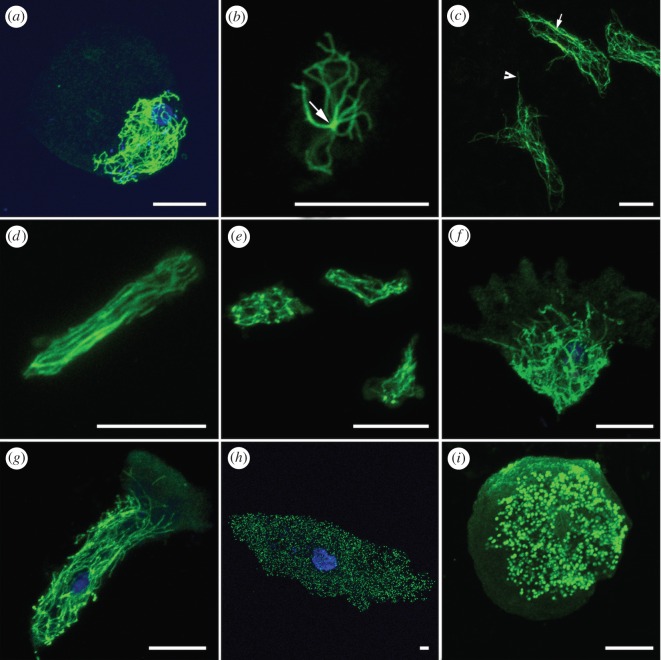
Confocal maximum intensity projection images showing MT architectures in diverse groups of amoebae. (*a*) *Vannella* sp. (*b*) *Dictyostelium discoideum.* Note MTOC, arrow. (*c*) *Acramoeba dendroida.* Note MT bundles on broader branch, arrow; and individual MT supporting the hair-like hyaline subpseudopodia, arrowhead. (*d,e*) *Hartmannella* sp. locomotive morphotype (*d*) and not actively moving amoeba (*e*). (*f,g*) *Flabellula citata.* Flat triangular locomotive morphotype (*f*) and elongated active locomotive morphotype (*g*). (*h,i*) Dot-like non-overlapping MT architectures in *Amoeba proteus* (*h*) and *Thecamoeba quadrilineata* (*i*). Microtubules (green) and DNA (blue). Scale bars, 10 µm.

**Figure 2.**
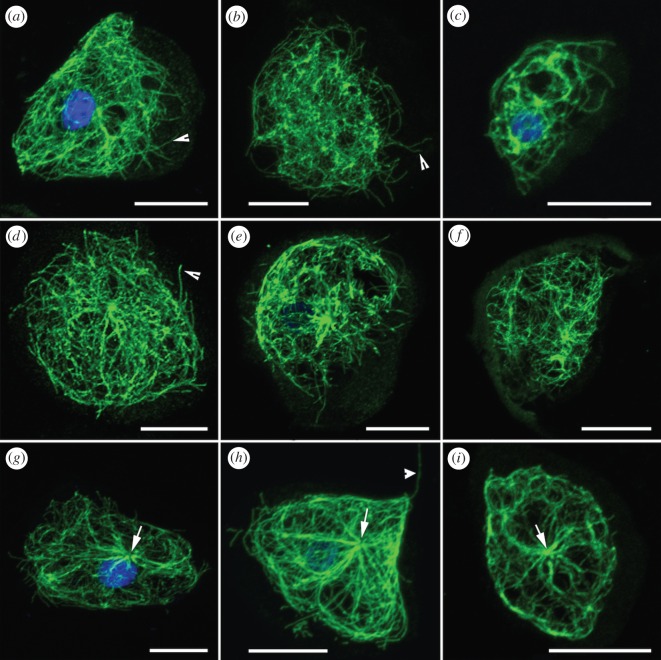
Confocal maximum intensity projection images showing MT architectures in *Cochliopodium*. Note all *Cochliopodium* spp. have one or more individual MTs in the hyaloplasm region as indicated in *a, b* and *d* panels by arrowheads. (*a*) *Cochliopodium pentatrifurcatum*. (*b*) *C. megatetrastylus.* (*c*) *C. minutoidum.* (*d*) *C. arabianum.* (*e*) *C. actinophorum*. (*f*) *C. spiniferum.* (*g*) *C. larifeili.* (*h*) Undescribed *Cochliopodium* sp. ‘crystal’. Note uroid supported by single MT, arrowhead. (*i*) *C. gallicum.* Note prominent MTOCs in (*g–i*), arrows. Microtubules (green) and DNA (blue). Scale bars, 10 µm.

Amoebozoa display a wide range of complex cytoplasmic MT architecture that differs in abundance, distribution, arrangement and formation of special structures such as the microtubule*-*organizing centre (MTOC). In general, the majority of the studied amoebozoans display cytoplasmic MTs in the form of long intertwined fibres distributed mainly within the bounds of the granular cytoplasm of the cell body (figures 1 and 2). Regions of cell periphery (hyaloplasm) in locomotive forms were mostly devoid of MT with the notable exception in *Cochliopodium* (figure 2). Amoebae with a fibrillar cytoplasmic MT form complex interwoven (three-dimensional network) fibres distributed throughout the granular cell body (figures 1*a*–*g* and 2). Two amoebae investigated belonging to the Tubulinea were observed to have MT architecture composed of long parallel arranged fibres in actively moving forms (figure 1*d*,*g*). The majority of the amoebae studied were not observed to possess distinguishable MT nucleation nodes (MTOCs), whereas we detected prominent MTOCs in two amoebae lineages, *Dictyostelium* (figure 1*b*) and three members of the genus *Cochliopodium* (figure 2*g*–*i*). Using the technique employed, two amoebae not shown to display fibrillar cytoplasmic MT include *Thecamoeba* and *Amoeba proteus*. ICC staining of cytoplasmic MT in whole mounts of these amoebae showed dot-like MT fibres that are uniformly distributed within the granular cell bodies (figure 1*h*,*i*). Detailed accounts of studied structures are given below.

#### 2.1.1. Vannella

The *Vannella* sp. examined here displays a very characteristic MT architecture limited to the granular cell body (figure 1*a*). The large hyaloplasm region of *Vannella* sp. is characteristically devoid of MTs (figure 1*a*). The MT's fibril network is dense and truncates or folds back into the granular cell body bordering the clear hyaloplasm region. Similar MT architecture was observed in the closely related amoeba, *Clydonella* (data not shown).

#### 2.1.2. Dictyostelium

*Dictyostelium discoideum* has the simplest cytoplasmic MT architecture than any of the amoebae studied here (figure 1*b*). MT fibres in *Dictyostelium* originate from a single perinuclear MTOC (figure 1*b*, arrow). The nucleation node is located in close proximity to the nucleus with the MT fibrils spreading out from the MTOC region to the rest of the cell body. Owing to their low number, individual MT fibres in *Dictyostelium* can be traced effectively, using a Z-stack (optical sections) imaging analysis. Using this technique, we observed some individual MTs to bifurcate, bend and sometimes criss-cross with one another to form a simple network (figure 1*b*). MTs originating from the MTOC were not observed to encircle or wrap around the nucleus.

#### 2.1.3. Acramoeba dendroida

*Acramoeba dendroida*, unlike most of the amoeba studied here, is a branching amoeba capable of forming fine hyaline, hair-like subpseudopodia that are rarely anastomosing. The cytoplasmic MT architecture of this amoeba is composed of long fibres of MTs running along the branching cell body and fine subpseudopodia (figure 1*c*). While most of the cytoplasmic MTs are arranged in parallel in this amoeba, some long MTs that are running diagonally, or slanted at various angles, can also be observed (figure 1*c*). In the thickest part of the cell branch, we observed some long MTs to bend or curve showing the appearance of an MT network, though less complex compared with other amoebae studied here. In addition, we observed one or more thick long bundles of MTs in the regions of the larger cell branches of the amoeba (figure 1*c*, arrow). Individual MTs support the hair-like hyaline subpseudopodia (figure 1*c*, arrowhead).

#### 2.1.4. *Hartmannella* and *Flabellula*

The two amoebae belonging to the clade Tubulinea that are shown here to display alternating MT architecture are *Hartmannella* sp. and *Flabellula citata* (figure 1*d*–*g*). These amoebae mostly display similar MT architecture observed in most flat-shaped amoebae such as *Cochliopodium,* where the cytoplasmic MTs are organized as an interweaving complex network uniformly distributed throughout the granular cell body (figure 1*e*,*f*). However, these amoebae were observed to display parallel arranged MTs when actively moving as in *Hartmannella* (figure 1*d*) or in the extended locomotive forms of *Flabellula citata* (figure 1*g*). While MT architecture of *Hartmannella* sp., when it is in non-directional movement, appears less characteristic with randomly overlapping MTs, the active locomotive form displays prominent MTs arranged in straight parallel arrays oriented in the direction of movement (figure 1*d*). *F. citata* has two locomotive morphotypes: broad triangular shape (figure 1*f*) and extended/elongated flat forms (figure 1*g*). The triangular flat locomotive form is the most common one and has similar MT architecture to most flat, disc-shaped amoebae, including *Cochliopodium* that displays few individual MTs extending past the granular cell body into the hyaloplasm region. Unlike *Cochliopodium, F. citata* has overall a less dense cytoplasmic MT network (figure 1*f*).

#### 2.1.5. *Amoeba proteus* and *Thecamoeba*

The MT staining of *Amoeba proteus* and two species of *Thecamoeba* resulted in unique and unusual cytoskeleton architecture (figure 1*h*,*i*). Unlike all of the amoebae studied here that form long fibrillar MTs, *Amoeba proteus* (figure 1*h*)*, Thecamoeba quadrilineata* (figure 1*i*) and an undescribed *Thecamoeba* sp. (data not shown) displayed strongly stained small MT dots in maximum projections of Z-stack serial images. The diameter of these individual (bundle) MT dots ranged from 0.25 to 1 µm in *Thecamoeba* and 0.25–1.5 µm in *Amoeba proteus.* Z-stack analyses of some specimens of these amoebae revealed that the MTs appear to grow in very short vertical fibres (data not shown).

#### 2.1.6. Cochliopodium

We collected the MT architecture data from ten species of *Cochliopodium* to evaluate the utility of cytoskeleton features in studying closely related lineages. All *Cochliopodium* species studied display fibrillar cytoplasmic MTs (figure 2). Species of *Cochliopodium* have a dense and complex cytoplasmic MT network within the granular cell body (figure 2). Unlike most of the amoebae examined in this study, species of *Cochliopodium* were also observed to have a characteristic presence of individual MTs extending out into the hyaloplasm region (figure 2, arrowheads). Most MT fibres in other amoebae are observed to truncate or curve back at the border of the granular cell body and hyaloplasm. However, in *Cochliopodium*, few individual fibres [1–8] are clearly observed to extend beyond the granular cell body. These individual MTs are present in regions of dense (folding) plasma membrane of the hyaloplasm or subpseudopodia (data not shown). The frequency of observation of this feature differed among *Cochliopodium* species. This feature was detected more often in *C. pentatrifurcatum, C. megatetrastylus* and *C. minutoidum* than the rest (figure 2*a*–*c*). In addition to this, we observed only single to a few MT fibres supporting uroidal projections (figure 2*h*), but this feature was only observed in those few cells with extended uroidal projections.

Other variation in MT architecture among *Cochliopodium* spp. included abundance and complexity of cytoplasmic MT fibres and detection of MTOC. While all *Cochliopodium* species examined have similar arrangement and density of granular cell body cytoplasmic MTs, abundance of MTs differed among species. The relatively larger species of *C. actinophorum* and *C. arabianum* have more dense and abundant MT networks than any of the other species studied (figure 2*d*,*e*). Data on MT architecture for *C. spiniferum* are limited (figure 2*f*). This is due, in part, to its adverse reaction to fixative agents and technical difficulties in retaining many cells until the end of the experiment. The most significant variation in MT architecture in *Cochliopodium* was the detection of prominent MTOC in three species, which include *C. gallicum, C. larifeili,* and an undescribed *Cochliopodium* sp. ‘crystal’ (figure 2*g*–*i*, arrows). These three species have MTOC in every cell examined including a unified MTOC in cells undergoing cellular fusion (data not shown). The MTOC in these species usually is located near the nucleus or at the midpoint of the cell body (figure 2*g*–*i*). However, the nucleus is not always closely associated with the MTOC. Similarly, MTs originating from the MTOC were not observed to wrap around the nucleus in any of these MTOC containing species. Owing to the abundance and complex MT network fibres, it was difficult to determine if all MTs in the cell originate from the single detectable MT nucleation node. MTs originating from the MTOC are observed to radiate from the dorsal cell surface of the cell to the rest of the cell body; however, it is not clear if all the MTs in the cell have one MTOC origin. On occasions, we observed an MTOC-like structure in a non-mitotic *Cochliopodium* sp., i.e. *C. megatetrastylus* (data not shown). However, the prominence and the frequency of this kind of MTOC-like structure was detected far less frequently than in the three species with prominent MTOCs.

### 2.2. Immunocytochemistry staining of actin in *Cochliopodium*

**Figure 3.**
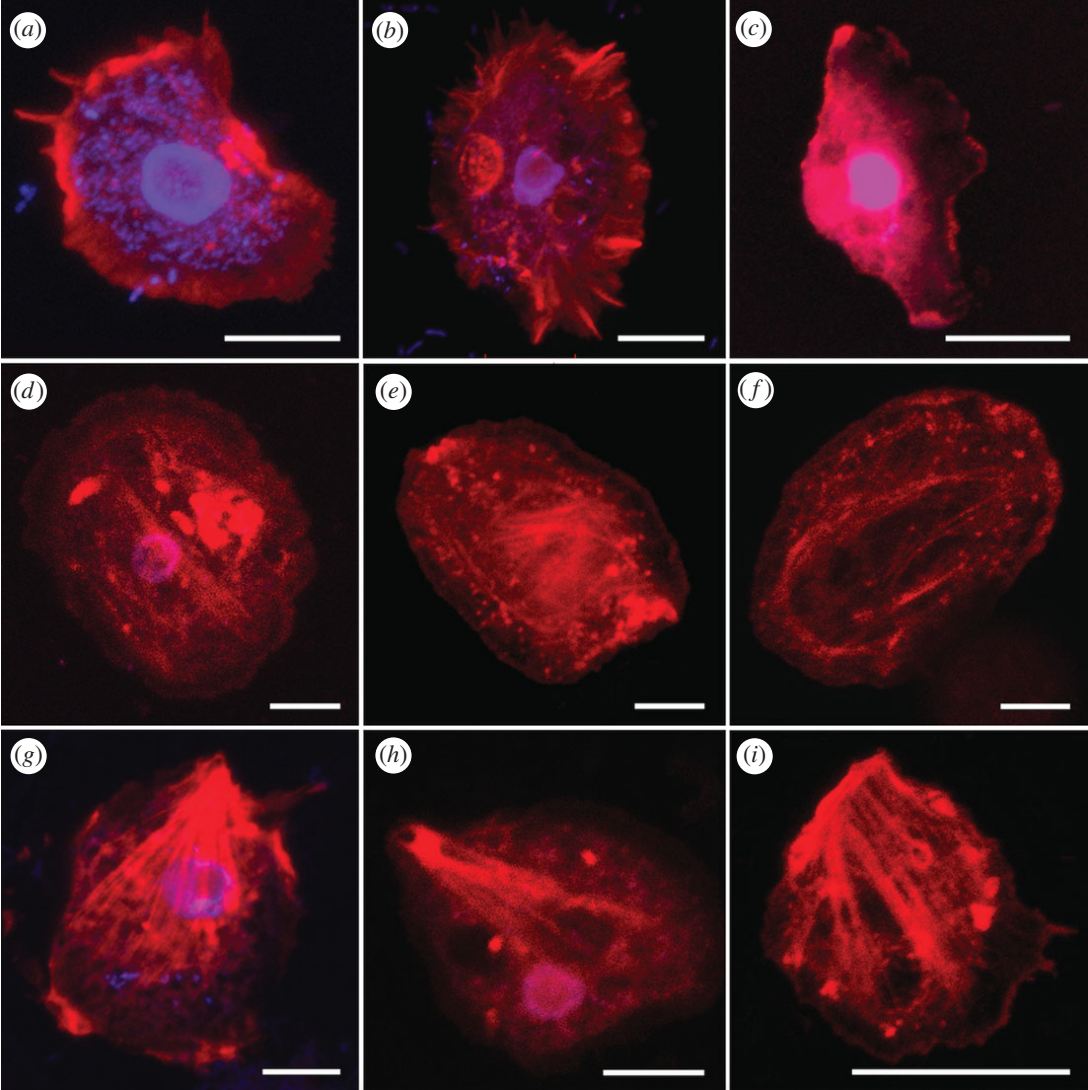
Confocal maximum intensity projection images showing F-actin architectures in *Cochliopodium*. (*a*) *Cochliopodium pentatrifurcatum*. (*b*) *C. minus.* (*c*) *C. minutoidum.* (*d*) *C. arabianum.* (*e,f*) *C. actinophorum.* (*g*) *C. larifeili.* (*h*) Undescribed *Cochliopodium* sp. ‘crystal’. (*i*) *C. gallicum.* F-actin (red) and DNA (blue). Scale bars, 10 µm.

We collected F-actin data from eight species of *Cochliopodium* (figure 3). While we have limited F-actin data on non-*Cochliopodium* species, our results showed that the examined amoebozoans express actin cytoskeleton throughout the cell body (data not shown). Most of our ICC staining of F-actin did not show visible microfilaments; it is rather shown as uniform staining differing in degrees of distribution/concentration (figure 3*c*). Detection of F-actin is usually higher in the leading edges (hyaloplasm) and posterior regions of the cell than the middle sections of the cell (figure 3). Some species are observed to express F-actin as bundles of microfibres or concentrated plaques of varying sizes (figure 3*d*,*e*,*h*,*i*). Observed variation of F-actin architecture among *Cochliopodium* spp. include (i) distribution, (ii) formation, and (iii) arrangement of microfibres. Based on these kinds of evidence, three categories of F-actin architecture can be recognized in *Cochliopodium*. The first category includes localization of a high, non-microfibrous actin concentration in the posterior and hyaloplasm regions of the cell, or within F-actin supported subpseudopodia. This type of architecture is found in *C. pentatrifurcatum, C. minus* and *C. minutoidum* (figure 3*a*–*c*). The second category includes the formation of bundles of microfilaments localized mainly in the central regions of the cell body. This type of arrangement is found in *C. actinophorum* and *C. arabianum* (figure 3*d*–*f*). The third category includes the formation of long straight bundle fibres, in the direction of movement, which radiate from posterior to anterior region of the cell in a triangular shape. This type of arrangement is found in *C. larifeili, C. gallicum* and undescribed *Cochliopodium* sp. ‘crystal’ (figure 3*g*–*i*).

## 3. Discussion

### 3.1. The nature of cytoskeleton architecture in Amoebozoa

The cytoskeleton is a hallmark of eukaryotic evolution; its molecular and morphological attributes are highly conserved and have played a pivotal role in our understanding of the origin and evolution of diverse eukaryotes [17,20–25]. Despite this, most studies of cytoskeleton architecture have been focused on non-amoeboid eukaryotic lineages or some amoeboids with a flagellate life cycle [35–38]. This is partly because most amoeboids, particularly those lineages with lobose type of pseudopodia, were considered simple, lacking complex cytoskeletal features and/or locomotory organelles [13]. There are very limited cytological studies of cytoskeleton in purely non-ciliated amoeboid lineages. Most of these studies are based on transmission electron microscopy (TEM) [30,32–34,39–41] and a handful on ICC techniques [31,32,42,43]. While these studies are only limited to a few lineages, and the techniques used had limitations (e.g. incompleteness and lack of immunologic confirmatory data in TEM studies), they indicated that some lobose amoebae are not completely devoid of cytoplasmic MTs [16,32–34,41]. The general consensus has been that the majority of amoebae belonging to Lobosa are assumed to lack cytoplasmic MTs, whereas the Conosa (Mycetozoa, Archamoebae) and Breviata are characterized by a conical and complex microtubular cytoskeleton, respectively [13,44]. Our study, for the first time, conclusively demonstrates that all amoebae, including diverse Lobosean and Variosean lineages, not only possess cytoplasmic MTs, but also a complex array of cytoskeleton architecture comparable to other non-lobosean lineages. These findings also provide strong evidence against claims that members of Lobosa lack complex cytoskeleton and cytoplasmic MTs.

The molecular and structural architecture of the eukaryotic cytoskeleton is highly conserved, as evidenced partially by the fact that we were able to successfully collect ICC data from amoebae using commercially available antibodies raised for mammals. We obtained consistent results for both actin (in the form of uniform staining, microfibre or bundles) and cytoplasmic MTs (in the form of regular fibrils or MTOC) using a protocol that was optimized for amoebae. However, MT staining in two distantly related amoebae, *Amoeba proteus* and *Thecamoeba,* resulted in a unique and unusual MT architecture. Cytoplasmic MT architecture in these amoebae appears microscopically as non-overlapping uniform dots giving it the appearance of upright MT fibres in cross-sectional viewing (figure 1*h*,*i*). The cytoskeleton of *Amoeba proteus* has been extensively studied using various techniques [27–30,39]. While the role and presence of F-actin in *Amoeba proteus* is well established [28,29], the presence and detection of cytoplasmic MTs has been controversial and inconclusive. Previous studies, using ICC techniques, were either unable to detect cytoplasmic MTs in *Amoeba proteus* [30,39] or found similar MT architecture as ours [31]. One study that used electron microscopy techniques also revealed four types of filaments in *A. proteus*, one of which falls in the size range known for MTs [30]. However, this study was unable to confirm the protein as an MT tubulin, using specific anti-tubulin antibodies. A similar study also detected MT only in mitotic cells [39]. These results have led to a conclusion that *A. proteus* lacked cytoplasmic MTs during the vegetative (interphase) stage [31,40]. Given we were able to successfully stain MTs in various groups of amoebae using our protocol and found similar MT architecture in a distantly related amoeba (*Thecamoeba*), our results are not likely artefactual, caused by some technical limitation. Rather, the preponderance of our evidence reveals a unique and novel cytoplasmic MT architecture in these amoebae. This result indicates that MT architecture in Amoebozoa might be more diverse than previously thought. Our finding further opens a new area of investigation on the role and evolutionary significance of MT in Amoebozoa in general and these lineages in particular.

### 3.2. Role of microtubule in amoeboid shape and locomotion

Our study demonstrates that cytoplasmic MT is an integral component of amoeboid morphology. In all cells examined, the majority of the granular cell region is occupied by a dense meshwork of interwoven MT fibrils. The arrangement of cytoplasmic MT is observed to change with the shape of the amoebae. Actively locomoting amoebae (locomotive morphotypes) have consistent MT architecture, whereas in non-directional movement, or otherwise, in non-motile states, MT architecture varies depending on the morphological/physiological state of the amoebae. This is more evident in amoebae such as *Hartmannella* and *Flabellula* that form dramatically different MT architectures in different physiological states. In the locomotive morphotypes of these amoebae, the MTs are notably long and arranged in parallel. While in other less active states of some elongated, non-tubular forms (e.g. *Hartmannella*) and alternative flat-shaped locomotive forms (*Flabellula*), they display loosely arranged overlapping MT networks (figure 1*f*). This finding has important phylogenetic significance for the clade Tubulinea, a taxon that encompasses these two lineages. The Tubulinea is primarily defined by molecular analysis [4,5]. Most of its members are characterized by tubular, subcylindrical pseudopodia but it also encompasses taxa with substantial morphological variations, including flat-shaped amoebae (e.g. *Flabellula*) and branching forms (e.g. *Leptomyxa*). These amoebae are capable of forming flattened subcylindrical pseudopodia under certain conditions [4]. Based on this, Smirnov *et al*. [4] defined Tubulinea as a group forming (or with the ability to form) tubular, subcylindrical pseudopodia and monoaxial cytoplasmic flow. Our finding reinforces this synapomorphic definition of Tubulinea by providing strong ICC data. That is alternative morphotypes of tubulinids, here exemplified by *Flabellula*, share similar MT architecture to other amoebae with tubular pseudopodia (*Hartmannella*) under certain physiological circumstances. This is in stark contrast to flat-shaped amoebae such as *Cochliopodium* or *Vannella* that were not observed to form parallel arranged MTs under any physiological conditions. This finding demonstrates the significance of ICC data in assessing homology of amoeboid structures in Amoebozoa (see below).

Mechanism of amoeboid movement and morphodynamic studies in Amoebozoa is poorly documented. Most studies related to this topic are based on a few lineages of amoebae, namely *Dictyostelium* [45], *Acanthamoeba* [42] and *A. proteus* [29]. It is generally believed that amoeboid movement in amoebae especially those with lobose pseudopodia are primarily driven by an actomyosin cytoskeleton [40]. This conclusion was reached based on the assumption that most lobose amoebae lack, or have poorly developed, MT cytoskeletons [40,46]. All amoebae examined here display a complex array of microtubular cytoskeletons in addition to presence of actin filaments throughout the cell body. Our study suggests that the MT cytoskeleton in amoebae might play more than a structural function in some amoebae, including a likely role in amoeboid movement. While MTs were localized within the granular cell area in most of the amoebae examined, some amoebae were notably observed to have MT-supported locomotory structures in other regions. These include regions of hyaloplasm and in the uroid in *Cochliopodium*; and branches, or fine hyaline regions, of *Acramoeba*. It could be deduced from this observation that the MT role in amoebae might differ by taxonomic groups. Preliminary results of a Colchicine treatment (a drug that inhibits MT polymerization) during an experiment in *Cochliopodium* shows that cell motility was greatly hampered by the use of the drug. Although the mechanism of amoeboid movement will require further experimentation involving cytomotive and related proteins (beyond the scope of this study), our results call for re-evaluation of our understanding of the role of MTs in Amoebozoan cell motility.

### 3.3. Evolutionary significance of cytoskeleton architecture in Amoebozoa

One of the major hurdles in Amoebozoa taxonomy has been paucity of morphological characters. Phenotypic characters generated using standard methods (light and electron microscopy), over the last two centuries, have reached their limits in defining novel relationships that are recovered in molecular phylogenies [4,8,10,12,47]. In this study, we introduce several new morphological characters related to the most taxonomically important feature, amoeboid structure, using an appropriate and novel technique. A list of potential cytoskeletal characters that could be mapped into phylogenetic interpretations, or used to build matrices for morphology-based analysis if more data are available, are listed in table 1. In this study, we have assessed the phylogenetic utility of collected ICC data in analysing deep and shallow nodes of Amoebozoa.

**Table 1.** Summary of observed variable ICC cytoskeleton characters in Amoebozoa.

microtubule cytoskeleton	
1	Presence or absence of MTOC—MTOC could be absent (i) or if present, it can vary in the following features. (ii) Prominent MTOC is detected in interphase cells at all time (e.g. *Dictyostelium*, figure 1*b; Cochliopodium,* figure 2*g–i*). (iii) Transient MTOC is rarely detected in few non-mitotic cells (e.g. some *Cochliopodium* species, data not shown).
2	Number of MTOC—based on previous published work [38] and this study the number of MTOC could vary from (i) one (figures 1*b* and 2*g–i*) to (ii) many within a single cell [38].
3	MTOC and its association with nucleus—variation of MTOC relative to the position of the nucleus may vary as follows. (i) MTOC is always closely associated to the nucleus (figure 1*b*). (ii) MTOC is not always closely associated to the nucleus (figure 2*g–i*). In this case, an MTOC can be observed separate from the nucleus within the cytoplasm.
4	MT arrangement originating from MTOC—variation in MT arrangement that radiate from the nucleation node of MTOC could vary in abundance. (i) Few MTs (figure 1*b*) or (ii) numerous MTs (figure 2*g–i*). Additionally, they could differ in arrangement in the following manner, (iii) distributed throughout the cell body (figures 1*b*, 2*g*–*i*) or (iv) mostly wrapped around the nucleus (unpublished data 2016). Variation also includes a combination of these characters.
5	Cytoplasmic MT could differ in arrangement including (i) loosely spaced network (*Flabellula,* figure 1*f*), (ii) tightly spaced network (figures 1*a* and 2), (iii) abundant (figure 2) or (iv) few (figure 1*b*).
6	Arrangement of MT in locomotive morphotypes—variation in MT arrangement including: (i) complex interwoven MT network (figures 1*a* and 2), (ii) MT arranged in parallel (figure 1*d,g*) or (iii) non-overlapping dot-like MT architecture (figure 1*h*,*i*).
7	Presence of MT in hyaloplasm and regions—variation was observed in detection of MT in the hyaloplasm regions. (i) On the leading hyaloplasm region (all *cochliopodiums*), (ii) uroid supported by single (figure 2*h,* arrowhead) or (iii) bundles (not shown) of MT.

actin cytoskeleton	
8	Actin distribution—variation in F-actin distribution in the cell area including: (i) non-fibrous uniform distribution throughout the cell (figure 3), (ii) high concentration of non-fibrous F-actin on leading edge of pseudopodia (figure 3*a*–*c*). Or (iii) high concentration of non-fibrous F-actin on the leading (anterior) and uroid (posterior) regions (figure 3*a*–*c*).
9	Nature of actin polymerization—variation in actin polymerization including: (i) plaque formation (figure 3*d*–*f*), (ii) Meshwork formation (figure 3*d*–*f*), (iii) bundle of microfilament formation (figure 3*g*–*i*).
10	Arrangement of actin microfilament—variation in microfilament including: (i) mostly located in centre of cell (figure 3*d*–*f*), (ii) microfilament supported subpseudopodia (figure 3*a*,*b*) or (iii) radiating microfilament bundles in the form of triangular shape (figure 3*g*–*i*).
11	Filament actin association with nucleus—F-actin can sometimes be observed (i) to closely associate, in high concentration, with nucleus (unpublished data 2016) or (ii) this feature is less prominent or absent (figure 3).

Our cytoskeletal data come from diverse amoebae representing major taxonomic groups in Amoebozoa: Tubulinea, Eudiscosea, Mycetozoa, Variosea and Himatismenida. A common feature shared among all these amoebae is the presence of complex cytoplasmic MTs contained within the granular cell body. Unlike previous classifications that distinguish two major taxonomic classes in Amoebozoa (‘Conosa’ and ‘Lobosa’) based on presence/absence of cytoskeleton [13], the newly collected data unites all amoebozoans as having a complex cytoplasmic MT array. Based on this result, it can be hypothesized the last common ancestor of Amoebozoa had a complex network of fibril MTs limited within the cell subcortex, thus conferring the unique adaptive features of amoeboid locomotion. It is evident from our results that most amoebozoans have kept the ancestral MT architecture, whereas some have evolved a derived MT architecture such as MT extending beyond the granular cell into the hyaloplasm region (figure 2). Alternatively, some lineages have independently evolved into a more dramatically different architecture in the form of non-overlapping, dot-like MT arrays (probably short vertically growing MTs, figure 1*h*,*i*). Therefore, we propose this ancestral MT architecture as a potential synapomorphy for Amoebozoa. Similar arguments can be made for the ancestral origin of the MTOC in Amoebozoa. Non-mitotic MTOCs have been described in various groups of amoebae, including members of Mycetozoa, Himatismenida and Centramoebida [10,12,16]. However, previously, none have been reported in Tubulinea or in most Eudiscosean lineages. The presence of an MTOC is also known in other eukaryotic cells [48]. The homology of MTOC in amoebae and other eukaryotic cells needs to be scrutinized before one can infer the ancestral origin of MTOCs in Amoebozoa or more generally in the eukaryotic tree of life.

Another cytoskeletal feature that can be used to define deep nodes within the Amoebozoa is the arrangement of MTs in locomotive morphotypes. One of the most stable and consistently recovered amoeba clade in molecular analysis is Tubulinea [4]. Despite the varied morphology observed in this clade, its members with drastic morphotypes share similar MT architecture (figure 1*d–g*). This finding corroborates both molecular phylogenies as well as morphological synapomorphy proposed by Smirnov *et al*. [4] for the group. The corroboration of our results with previously reported molecular and redefined light microscopy data makes cytoskeletal characters very promising for studying Amoebozoa evolution at deeper relationships. Additional cytoskeletal characters that could potentially be used to define deeper nodes in amoebae include presence of individual MTs in hyaloplasm and general complexity of MT networks in taxonomic groups such as *Cochliopodium* or other related flat-shaped amoebae. However, additional data representing major lineages of amoebae are necessary for comprehensive assessment of these cited attributes, and other characters listed in table 1.

In order to assess utility of cytoskeletal characters in shallow nodes, we collected ICC data from a monophyletic genus, *Cochliopodium*, whose phylogeny is fairly known [49–51]. Interestingly, we find enough variability both in actin and MT architectures among *Cochliopodium* spp. to generate evidence for, and evaluate robustness of, these cytoskeletal characters (table 1) based on the published molecular phylogenetic tree. All *Cochliopodium* species have a dense MT network and share the presence of individual MTs in the hyaloplasm regions. The presence of MTs in the hyaloplasm was more frequently detected in three sister taxa including *C. pentatrifurcatum, C. minus* and *C. minutoidum* than the remaining *Cochliopodium* species. In addition, the sister relationship of these three taxa is supported by a high concentration of F-actin at the periphery of the cells. *C. pentatrifurcatum* and *C. minus,* two genetically indistinguishable sister lineages [52] share a similar subpseudopodial actin architecture supported by bundles of actin microfilaments (figure 3*a*,*b*).

Another two well-supported *Cochliopodium* sister taxa, in molecular phylogenies, that are observed to share unique actin architecture are *C. actinophorum* and *C. arabianum.* Both amoebae have bundles of actin microfilaments mostly located in the centre of the cell. In addition to this, both amoebae have a denser MT network compared with other species examined. However, the density of the MT network in these amoebae may be more reflective of their relative size, rather than a distinctive morphological character.

Finally, the two least understood lineages of *Cochliopodium, C. larifeili, C. gallicum* and an undescribed *Cochliopodium* sp. ‘crystal’ share characteristic cytoskeleton architecture. In *Cochliopodium,* one of the most reliable characters for species identification and for the whole genus is the morphology of the dorsal surface microscale [53]. Unlike most described *Cochliopodium* spp. that have tower-like surface microscales, *C. larifeili* and *C. gallicum* have a markedly different, non-conventional scale morphology. This puts them at odds morphologically with the other members of the genus. Their placement in the molecular phylogenetic tree is not fully analysed or resolved. In one study, they group as sister taxa basal to the rest of the *Cochliopodium* spp. [52]*.* The undescribed *Cochliopodium* sp. ‘crystal’ forms a strongly supported sister group relationship with *C. larifeili* in COI gene phylogenetic analysis (unpublished data 2016). We find robust cytoskeletal architecture both in F-actin and MT supporting the relationship of these three species, which include a prominent MTOC (figure 2*g–i*) and triangular-shaped radiating bundles of actin microfilament (figure 3*g*–*i*). Despite differences in size (*C. larifeili* almost twice the size of *C. gallicum* and *C.* sp. ‘crystal’ of a median size), the three amoebae uniquely express strongly stained bundles of F-actin, not observed in any amoeba examined.

Additionally, these three species have prominent MTOCs detected consistently in all specimens examined for each of the species. MTOC or MTOC-like structures were observed in a few specimen preparations of *C. megatetrastylus* (data not shown). Even if most specimens of *C. megatetrastylus* examined were devoid of MTOC, this result raises the possibility that some *Cochliopodium* spp. might be capable of forming MTOCs during their interphase cycle. Other *Cochliopodium* spp. reported to possess MTOC based on TEM studies include *C. plurinucleolum* [54], *C. spiniferum* [55] and *C. barki* [56]. ICC data for *C. barki* and *C. plurinucleolum* are not available, but our results do not reveal a prominent MTOC in *C. spiniferum* as observed in the three sister taxa (figure 2*f*). In original descriptions of *C. larifeili* and *C. gallicum,* no MTOC or microtubular structures were reported, though Kudryavtsev & Smirnov [57] report bundles of microfilament in *C. gallicum.* These mixed reports lead us to believe that TEM preparations are not optimal for the study of cytoskeletal architecture in many Amoebozoa. Among other technical limitations, cytoskeletal architecture based on TEM images could be a hit or miss case of chance observations, because the complex structure of MTs usually are not detected in a single serial section. Similarly, it is difficult to predict the nature of MTs (mitotic versus cytoplasmic) from randomly selected sections, in addition to the possible TEM fixation artefacts. On the contrary, ICC and confocal microscopy methods are superior to TEM imaging, because it enables analysis of sequential sections in an intact whole mount specimen concurrent with the use of specific antibodies to target the desired protein. Even if it is likely that some *Cochliopodium* spp. might be able to form transient cytoplasmic MTOCs during their life cycle, the consistent detection of MTOCs in *C. larifeili, C. gallicum* and *C.* sp. ‘crystal’ can be considered as a unique shared character supporting their close relatedness in genetic analysis.

## 4. Material and methods

### 4.1. Taxa studied

We studied diverse groups of amoeboid lineages representing all major subclades of Amoebozoa [12,18]. These include flat-shaped amoeba belonging to Eudiscosea (*Vannella* sp. and *Thecamoeba quadrilineata*); 10 members of clade Himatismenida: *Cochliopodium pentatrifurcatum*, *C. minus* CCAP1437/1A, *C. megatetrastylus, C. minutoidum*, *C. actinophorum, C. arabianum, C. spiniferum, C. larifeili, C. gallicum* and an undescribed *Cochliopodium* species, labelled ‘crystal’ owing to presences of unique large cytoplasmic inclusions, shown to form a close relationship with *C. larifeili* in genetic analysis (unpublished data 2016); three tubulinids: *Hartmannella* sp.*, Flabellula citata* and *Amoeba proteus;* a variosean, *Acramoeba dendroida* and a mycetozoan, *Dictyostelium discoideum.* These taxa were selected based on taxonomic coverage and their availability. Cultures were maintained at Spelman College in ATCC medium 997 for freshwater/marine amoebae, with mixed bacteria as food. For ICC studies, all amoebae were further subcultured in Petri dishes with bottled natural spring water (Deer Park®, Nestlé Corp., Glendale, CA) or artificial seawater (Instant Ocean®, Blacksburg, VA), for *F. citata,* with added autoclaved grains of rice. Cultures of *D. discoideum* and *A. proteus* were obtained from WARD'S Natural Science Establishment (Rochester, NY).

### 4.2. Immunocytochemistry

Subcellular structures including cytoskeleton (F-actin and MT) and DNA of whole mount amoeba cells were studied using ICC methods. Adherent amoebae grown in two-well glass chamber slides (Thermo Scientific™, Nunc Lab-Tek, Rochester, NY) in a medium of bottled Deer Park water (artificial seawater), and grains of rice were rinsed with fresh medium first and then fixed in −80°C cooled methanol for 2 min. For actin, amoebae were fixed in 3% formaldehyde (Ladd Research, Williston, VT) for 1–5 min and permeabilized in phosphate-buffered saline (PBS) containing 0.5% Triton X-100 for 3 min. After incubation, all non-specific binding was blocked with 1% bovine serum albumin (BSA) for 10 min. Staining of F-actin fibres with Alexa Fluor 594® phalloidin (Life Technologies; 1 : 1000) was performed for 1 h at room temperature or overnight at 4°C. Microtubules were stained first with primary mouse anti-alpha-tubulin monoclonal antibody (Life Technologies; 1 : 500) for 1 h and a secondary Alexa Fluor® 488 rabbit anti-mouse IgG (H + L) for 45 min. DNA was stained with Hoechst 33 358 (1 : 1500) for 5 min. After rinsing in PBS, amoebae were mounted in a drop of fluoromount-G (Southern Biotech) under a coverslip. The whole mount preparations fixed in glass slides were examined with a Zeiss LSM 700 Inverted Confocal Microscope (Carl Zeiss MicroImaging, Gottingen, Germany). Well-preserved specimens (more than 100 cells for each species), primarily focusing on locomotive morphotypes, were viewed either from top or bottom side of the cells. Usually, 6–20 optical sections 0.1–0.38 µm thick were obtained while scanning through the specimen using the Z-stack option in ZEN microscope software (Carl Zeiss v. 5.5, Heidelberg, Germany). The maximum intensity projection option was used to make reconstructions from all adjacent optical sections in a series.

## Data accessibility

We have no supplementary material in this study. Our data are consistent and selected images representative of each taxon are included in the results.
